# Benchmarking surgical infections: A five years NSQIP-based analysis of postoperative infectious morbidity in general surgery at a tertiary care hospital in Pakistan

**DOI:** 10.12669/pjms.42.(11AASC).15659

**Published:** 2026-04

**Authors:** Narmeen Asif, Nargis Maqbool, Abdul Rehman Alvi

**Affiliations:** 1 Dr. Narmeen Asif, Department of Surgery, Aga Khan University Hospital, Karachi, Pakistan; 2 Dr. Nargis Maqbool, Department of Surgery, Aga Khan University Hospital, Karachi, Pakistan; 3 Dr. Abdul Rehman Alvi, Department of Surgery, Aga Khan University Hospital, Karachi, Pakistan

**Keywords:** Benchmarking, General surgery, Low- and middle-income countries, NSQIP, Postoperative morbidity, Quality improvement, Surgical site infection

## Abstract

**Background & Objectives:**

Surgical site infections (SSIs) remain a leading cause of postoperative morbidity in low- and middle-income countries (LMICs). This study evaluates five years trends in postoperative infectious complications in general surgery using American College of Surgeons NSQIP data from a tertiary care hospital in Pakistan.

**Methodology:**

Retrospective analysis of 2,650 consecutive adult patients undergoing general surgical procedures from January 2020 to December 2024 at the Department of General Surgery, Aga Khan University Hospital, Karachi. Standard NSQIP variables and risk-adjusted outcomes were examined. Predictors of SSI were identified using multivariable logistic regression, and institutional performance was benchmarked via observed-to-expected (O/E) ratios.

**Results:**

The overall SSI rate was 8.0% (superficial, 4.9%; deep, 0.3%; organ/space, 2.8%), with the highest rates following hepatopancreaticobiliary (19.6%), colorectal (18.3%), and small-bowel procedures. Independent predictors of SSI were open surgical approach (OR 3.42, 95% CI 2.16–5.43), operative time >120 minutes (OR 2.76, 95% CI 1.94–3.91), ASA class III–IV (OR 1.61, 95% CI 1.15–2.24), and diabetes mellitus (OR 1.47, 95% CI 1.03–2.11). The annual SSI rate declined significantly from 9.1% in 2020 to 6.2% in 2024 (p=0.031), and overall 30-day morbidity fell from 10.7% to 6.9% (p<0.001). Risk-adjusted morbidity improved from an O/E ratio of 1.09 in 2020 to 0.76 in 2024 (better than expected), while mortality O/E remained near 1.0 throughout.

**Conclusion:**

Despite initially elevated SSI rates compared with international NSQIP benchmarks, sustained participation in NSQIP was associated with significant reductions in infectious morbidity and achievement of better-than-expected overall outcomes. Expansion of minimally invasive surgery and reduction in operative time represent key opportunities for further improvement in LMIC settings.

## INTRODUCTION

Surgical site infections (SSIs) and other postoperative infectious complications remain a major source of morbidity following general surgery.^1^ The burden is particularly high in low- and middle-income countries (LMICs), where constrained resources, delayed patient presentations, and a greater reliance on open surgical procedures contribute to elevated infection rates.^2^,^3^ Each year, an estimated 313 million surgical procedures are performed worldwide, yet surgical conditions continue to account for a substantial share of preventable morbidity and mortality, which are more common in LMICs.^4^ In these settings, SSIs not only prolong hospital stay and increase readmission rates but also impose significant financial strain on already fragile healthcare systems.^5^

The American College of Surgeons National Surgical Quality Improvement Program (ACS NSQIP) is a validated, risk-adjusted, outcomes-based registry that has led to meaningful improvements in surgical care.^6^ Originally derived from the Veterans Affairs NSQIP, which achieved a 47% reduction in 30-day postoperative mortality and a 43% reduction in morbidity, the program has demonstrated similar effectiveness across the private sector. Today, NSQIP enables participating hospitals worldwide to benchmark risk-adjusted outcomes against international standards.^7^,^8^ Through systematic collection of more than 150 preoperative, intraoperative, and 30-day postoperative variables, NSQIP provides a robust platform to identify performance gaps and guide targeted quality-improvement interventions.^9^

Despite its widespread adoption in high-income countries, NSQIP participation in LMICs remains limited. Aga Khan University Hospital (AKUH) in Karachi, Pakistan, is among the earliest LMIC institutions to join ACS NSQIP and has previously reported outcomes in neurosurgery and Orthopaedics, highlighting both strengths (e.g., low venous thromboembolism rates) and areas requiring improvement (e.g., sepsis and SSIs).^10^,^11^ However, comprehensive NSQIP-based analyses of general surgery outcomes at AKUH, focusing specifically on postoperative infectious morbidity, have been lacking.

Although AKUH has been an active ACS NSQIP participant for several years, detailed assessments of general surgery outcomes- especially postoperative infectious complications, are lacking. Prior institutional NSQIP-based reports have primarily focused on neurosurgery and Orthopaedics, leaving a gap in understanding infectious morbidity patterns, modifiable risk factors, and longitudinal quality trends in general surgical practice. This study addresses this gap by examining five years of consecutive NSQIP data (2020–2024) with a specific focus on surgical site infections and other postoperative complications, identifying potentially modifiable risk factors, and comparing our risk-adjusted outcomes with international NSQIP benchmarks.

### Primary Objective:

To determine the incidence and pattern of surgical site infections in general surgery patients using institutional NSQIP data from 2020 to 2024

### Secondary Objectives:

- To assess other postoperative outcomes and 30-day morbidity & mortality over the five years
- To identify patient-related and surgical factors associated with increased risk of SSI
- To assess year-wise trends in postoperative outcomes over the five-year period
- To compare institutional infection-related outcomes with international NSQIP benchmarks

## METHODOLOGY

A retrospective cohort study was conducted at the Department of General Surgery, Aga Khan University Hospital, Karachi, Pakistan, utilizing institutional ACS-NSQIP data from January 2020 to December 2024. Non-probability consecutive sampling of all NSQIP-eligible general surgical procedures.

### Ethical considerations:

Ethical Review Committee exemption was obtained from Aga Khan University, Karachi (ERC No: 2025-11745-36589; dated September 23, 2025.). The study involved analysis of de-identified secondary data from the institutional NSQIP database, with no direct patient contact or intervention. Data confidentiality was maintained in compliance with institutional and NSQIP data protection standards

### Study criteria:

All consecutive adult patients (≥18 years) who underwent inpatient general surgical procedures recorded in the NSQIP database between 2020 and 2024 were included. Standard ACS-NSQIP exclusion criteria were applied, including patients younger than 18 years, those with ASA class VI, trauma cases, transplant procedures, HIPEC cases, concurrent procedures, and operations occurring within 30 days of a previous NSQIP-captured surgery at the same hospital. In addition to these, we applied study-specific exclusions for procedures involving breast or endocrine surgery, as well as already infected interventions such as incision and drainage, debridement, and abdominal washouts performed for collections.

### Data collection and Variables:

Data was extracted by trained and certified Surgical Clinical Reviewers (SCRs) using the standardized ACS NSQIP eight days cycle sampling strategy to minimize selection bias. The institutional NSQIP dataset contains over 150 standardized variables, from which we selected those relevant to this study. Variables of interest included demographic characteristics (age, sex, BMI, and comorbidities such as diabetes, hypertension, smoking status, and immunosuppression), perioperative factors (ASA class, operative duration, surgical approach, urgency of procedure, intraoperative blood transfusion, and procedure type), and 30 days postoperative outcomes. Postoperative outcomes assessed included infectious morbidity (superficial, deep, and organ-space SSIs, pneumonia, and urinary tract infection), systemic complications (DVT, PE, and myocardial infarction), length of hospital stay, readmissions, unplanned return to the operating room, and overall, 30-day morbidity and mortality.

### Statistical analysis:

Data was analyzed using standard descriptive and inferential statistics. Continuous variables were summarized as mean ± standard deviation (SD) or median with interquartile range (IQR) and compared using the t-test or one-way ANOVA for normally distributed data, and the Mann–Whitney U or Kruskal–Wallis test for non-normal data. Categorical variables were compared using the Chi-square test or Fisher’s exact test. Year-wise trends (2020–2024) were assessed using the Linear-by-linear association test for trend. Institutional outcomes were compared with NSQIP benchmarks using observed-to-expected (O: E) ratios. Predictors of postoperative infectious morbidity were evaluated using logistic regression. Variables with p < 0.20 on univariate analysis and clinically relevant covariates were included in the multivariate model to avoid missing potentially important confounders. Adjusted odds ratios (aOR) with 95% confidence intervals (CI) were reported, with p < 0.05 considered statistically significant. All analyses were performed using SPSS Statistics version 25.0.

## RESULTS

### Baseline Characteristics:

A total of 2,650 patients undergoing general surgical procedures between 2020 and 2024 were included. The mean age was 48.6 ± 16.3 years, and 54.6% were males. The mean BMI was 27.3 ± 6.4 kg/m². Key patient-related risk factors included diabetes mellitus (17.3%), smoking (7.9%) and immunosuppression (1.0%). Most patients were classified as ASA I-II (71%) (Table-I).

**Table 1:** Baseline characteristics of study cohort (2020-2024, n=2,650).

Variable	Definition/Categories	n (%) or mean ± SD
***Age (years)***		
	(mean ± SD)	48.63 ±16.31
	<60 years	1895 (71.6%)
	≥60 years	755 (28.4%)
***Gender***		
	Male	1446 (54.6%)
	Female	1204 (45.4%)
***BMI (kg/m^2^)***		
	(mean ± SD)	27.32 ± 6.35
	Underweight <18.5	183 (6.9%)
	Normal weight 18.5-24.9	969 (36.6%)
	Overweight 25-29.9	866 (32.7%)
	Obese ≥30	632 (23.8%)
***Comorbidities/ Risk factors***		
Diabetes	Present	458 (17.3%)
	Absent	2192 (82.7%)
Hypertension	Present	767 (28.9%)
	Absent	1883 (71.1%)
Smoking	Yes	210 (7.9%)
	No	2440 (92.1%)
Immunosuppression	Yes	26 (1.0%)
	No	2624 (99%)
***ASA Classification***		
	ASA I-II	1881 (71%)
	ASA III-IV	769 (29%)

### Operative characteristics:

**Fig.1:**
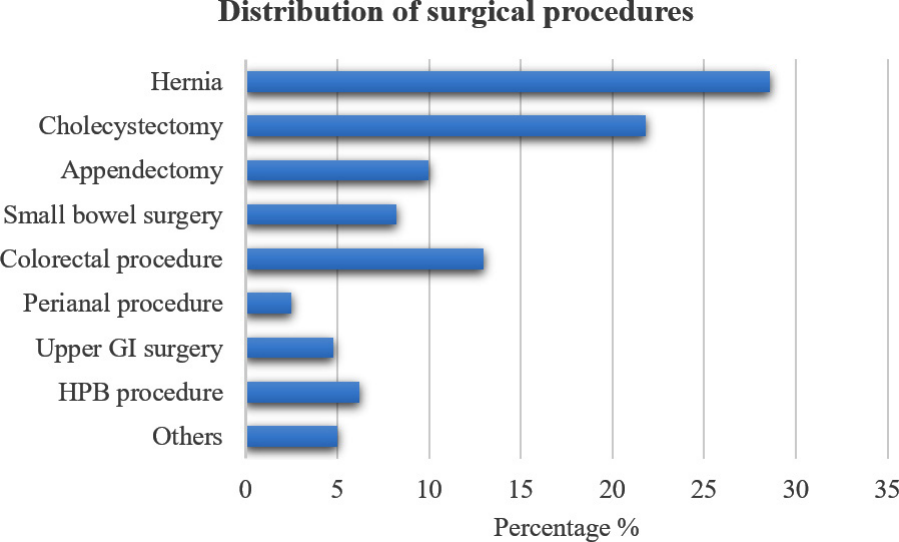
Distribution of surgical procedures.

Most procedures were elective (73.9%), performed under general anesthesia (98.2%). Open surgery accounted for 53.6% of procedures, with laparoscopic surgery in 46.4%. The median operative duration was 95 minutes (IQR 67–144 minutes). Intraoperative blood transfusion was required in 6.4% of patients (Table 2). The most common procedures were hernia repair (28.6%), cholecystectomy (21.8%), and colorectal procedures (13%) as illustrated in Fig.1.

**Table-II:** Operative Characteristics.

Type of procedure	Elective	1958 (73.9%)
	Emergency	692 (26.1%)
Surgical approach	Laparoscopic	1230 (46.4%)
	Open	1420 (53.6%)
Anesthesia	General	2603 (98.2%)
	Regional	42 (1.6%)
	Local	5 (0.2%)
Intraoperative blood transfusion	Yes	169 (6.4%)
	No	2481 (93.6%)
Duration of surgery	Minutes (Median (IQR))	95 mins (67-144)

### Incidence of Surgical Site Infection (SSI):

The overall SSI rate was 8%. Among these, superficial infections accounted for 4.9%, deep infections for 0.3%, and organ/space infections for 2.8%. SSI was most frequently observed following hepatopancreaticobiliary (HPB) (19.6%), colorectal (18.3%) and small bowel procedures (18.1%). Table-III outlines the incidence and type of SSI according to surgical procedure.

**Table-III:** Incidence and type of SSI by Surgical procedure.

Procedure	n	SSI n (%)	Superficial SSI	Deep SSI	Organ/Space SSI
Hernia	758	25 (3.3%)	20 (2.6%)	-	5 (0.7%)
Cholecystectomy	579	13 (2.2%)	10 (1.7%)	-	3 (0.5%)
Appendectomy	265	13 (4.9%)	7 (2.6%)	1 (0.4%)	5 (1.9%)
Small bowel surgery	216	39 (18.1%)	25 (11.3%)	-	14 (6.5%)
Colorectal procedures	345	63 (18.3%)	39 (11.3%)	5 (1.4%)	19 (5.5%)
Perianal procedures	65	1 (1.5%)	1 (1.5%)	-	-
Upper GI surgeries	127	13 (10.2%)	7 (5.5%)	-	6 (4.7%)
HPB procedures	163	32 (19.6%)	11 (6.7%)	1 (0.6%)	20 (12.3%)
Others*	132	12 (9.1%)	9 (6.8%)	-	3 (2.3%)
Overall SSI	2650	211 (8.0%)	129 (4.9%)	7 (0.3%)	75 (2.8%)

* Other procedures include diagnostic laparoscopy and biopsy, retroperitoneal tumor excision, lymphadenectomy, mesenteric mass excision, soft tissue procedures and umbilectomy.

### Postoperative outcomes:

The median length of hospital stay was two days (IQR 1-5 days). Other postoperative complications included pneumonia (0.8%), urinary tract infection (0.6%), myocardial infarction (0.6%), DVT (0.1%), and pulmonary embolism (0.0%). Readmissions occurred in 5.1% of patients, with 3.9% due to the primary surgical condition. Unplanned return to the operating room was observed in 2.1% of patients. Overall, 30-day morbidity was 9.2% and 30-day mortality 1.7% (Table-IV).

**Table-IV:** Postoperative outcomes.

Variable	n (%)
***Other Post-operative Complications***	
***Pneumonia***	
Urinary tract infection (UTI)	22 (0.8%)
Myocardial infarction	15 (0.6%)
Deep venous Thrombosis (DVT)/	15 (0.6%)
Pulmonary embolism (PE)	2 (0.1%)
Readmissions	136 (5.1%)
Unplanned return to OR	56 (2.1%)
30 days overall morbidity	245 (9.2%)
30 days mortality	46 (1.7%)

### Predictors of SSI:

On univariate analysis, age ≥60 years, diabetes mellitus, higher ASA class, open surgery, operative duration >120 minutes, and intraoperative blood transfusion were significantly associated with SSI (all p < 0.05). In multivariate logistic regression, independent predictors of SSI were diabetes mellitus (adjusted OR 1.47, 95% CI 1.03–2.11; p = 0.04), ASA class III–IV (adjusted OR 1.61, 95% CI 1.15–2.24; p = 0.006), open approach (adjusted OR 3.42, 95% CI 2.16–5.43; p < 0.001), and operative time >120 minutes (adjusted OR 2.76, 95% CI 1.94–3.91; p < 0.001) (Table-V).

**Table-V:** Univariate and multivariate analysis of factors associated with SSI.

Variable	Univariate Analysis	Multivariate Analysis	
	p-value	p-value	Odds ratio 95% CI
Age ≥60 years	0.002	0.90	0.98 (0.70-1.37)
Gender	0.870		
BMI ≥30	0.115	0.73	1.03 (0.87-1.23)
Diabetes Mellitus	<0.001	0.04	1.47 (1.03-2.11)
Smoking	0.384		
Immunosuppression	0.160	0.44	1.56 (0.51-4.79)
ASA III-IV	<0.001	0.006	1.61 (1.15-2.24)
Open approach	<0.001	<0.001	3.42 (2.16-5.43)
Emergency procedure	0.629		
Intraoperative blood transfusion	<0.001	0.667	1.26 (0.82-1.95)
Duration of surgery >120minutes	<0.001	<0.001	2.76 (1.94-3.91)

### Year-wise Trends (2020–2024):

**Fig.2:**
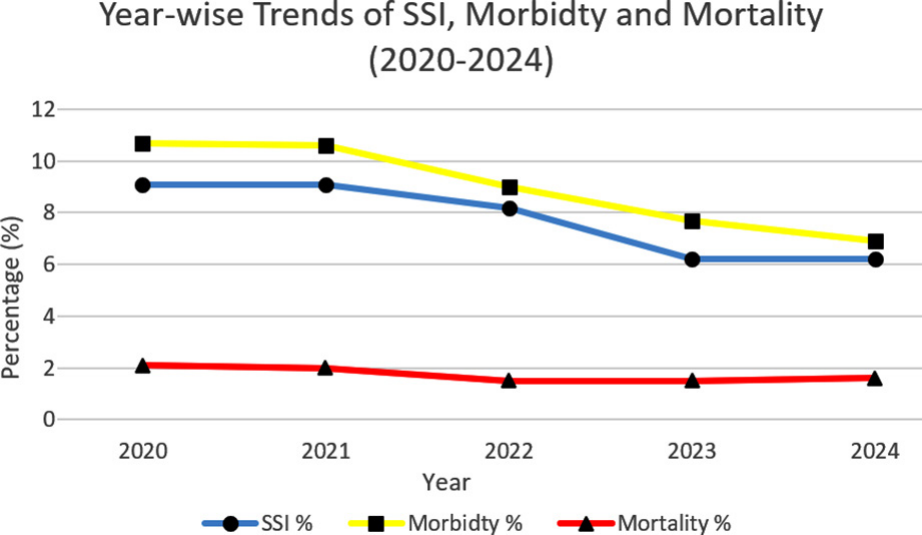
Line graph showing year-wise trends (2020–2024).

Year-wise outcomes are summarized in Table-6 and illustrated in Fig.2. The annual SSI rate decreased from 9.1% in 2020 to 6.2% in 2024 (p=0.031). Overall, 30 days morbidity decreased from 10.7 to 6.9% over the same period (p<0.001). 30-day mortality remained stable (range 1.5-2.1%, p=0.916).

**Table-VI:** Year-wise trends of postoperative outcomes.

Year	n	SSI n (%)	30 days morbidity n (%)	30 days mortality n (%)
2020	607	55 (9.1%)	65 (10.7%)	13 (2.1%)
2021	605	55 (9.1%)	64 (10.6%)	12 (2%)
2022	589	48 (8.2%)	53 (9%)	9 (1.5%)
2023	530	33 (6.2%)	41 (7.7%)	8 (1.5%)
2024	318	20 (6.2%)	22 (6.9%)	5 (1.6%)

### Benchmarking Against NSQIP Expected Rates:

**Fig.3:**
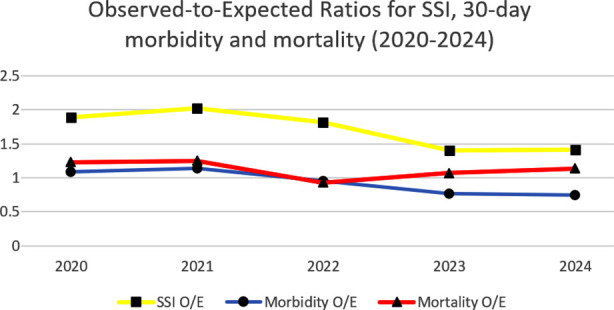
Benchmark comparison.

When compared with risk-adjusted NSQIP expected rates, the observed-to-expected (O/E) ratio for SSI was consistently above 1.0 during the study period, peaking at 1.99 in 2021, but demonstrated a downward trend in subsequent years. The O/E ratio for overall 30-day morbidity improved from 1.09 in 2020 to 0.76 in 2024, indicating performance better than expected by the end of the study period. Mortality O/E ratios remained close to unity throughout (range 0.94–1.25) with no significant directional trend (Fig.3).

## DISCUSSION

In this study, surgical site infections (SSIs) occurred in 8% of our general surgery patients over the five-year period, which is higher than the rates typically reported through NSQIP internationally. However, the key finding is the clear and steady improvement over time i.e.: SSI rates fell from 9.1% in 2020 to 6.2% in 2024, and overall complications dropped even more sharply. By 2024, our risk-adjusted morbidity was better than NSQIP expectations (O/E 0.76), while mortality remained consistent with international benchmarks. These findings of starting higher but improving faster than predicted is encouraging for a high-volume tertiary hospital in Pakistan.

Our SSI rate is comparable to what many centers in low- and middle-income countries report. Studies from Africa and Asia commonly show SSI rates between 7% and 15% for general surgery, often due to greater reliance on open surgery and longer operative durations.^12^,^13^ We saw a similar pattern as more than half of our operations were open procedures, and both open surgery and operations lasting longer than two hours were the strongest predictors of infection. Diabetes and higher ASA class also increased risk, although not as strongly as operative technique and duration. This is consistent with large international analyses and suggests that, in our context, expanding minimally invasive surgery and reducing operative time may yield greater improvement than solely targeting patient-level risk factors.^4^

Our five-year continuous analysis using a single, standardized NSQIP methodology was able to demonstrate sustained quality improvement within the same institution over time. The reduction in overall morbidity from slightly above expected performance to clearly better than expected shows that regular measurement, feedback, and small, incremental changes can produce meaningful results even in resource-constrained environments. The early peak in SSI O/E in 2021 likely reflects the operational disruptions of the COVID-19 period, including staffing challenges, higher proportion of emergency procedures and altered theatre workflows,^14^,^15^ but the subsequent downward trend suggests that the system adapted quickly.

Mortality remained close to NSQIP predictions throughout the entire period, which is important. It indicates that our improvements in morbidity were not achieved by avoiding high-risk cases or shifting the case mix. We continued operating on the same types of patients but produced better outcomes.

Although no single formal intervention or standardized bundle was introduced during the study period, regular NSQIP feedback and institutional audits informed incremental practice modifications, including increased adoption of minimally invasive approaches over the years and heightened attention to operative efficiency and infection-prevention practices.

Overall, our results indicate that NSQIP can be implemented effectively in a Pakistani academic hospital, achieving improvements like those seen in major U.S. centers.^3^,^5^ This study offers local evidence that consistent outcome measurement, regular audit, and comparison against external standards can drive real progress. For hospitals in comparable settings, the message is to build a dependable system for data collection, expand minimally invasive surgical options, work toward shorter operative times, and better outcomes are likely to follow. It provides local evidence that honest, structured outcomes tracking and benchmarking can drive improvement.

### Limitations:

This study is limited by its retrospective, single-center design, which restricts causal inference and generalizability. Although ACS NSQIP provides high-quality, standardized data, outcomes are limited to 30-day follow-up, and some procedure-specific details and institutional process measures are not captured, which may influence infectious risk. Additionally, NSQIP case sampling may limit detailed analyses of low-volume procedures.

### Author`s Contribution:

**NA:** conception and design of the study, data acquisition, interpretation and manuscript writing. **NM:** data acquisition, statistical analysis and interpretation, and manuscript writing. **ARA:** conception and design of the study, overall supervision, and final responsibility for the integrity and accuracy of work. All authors have approved the final version of the manuscript and agree to be accountable for all aspects of the work.
